# Institutional Integration and Risk-Based Food Safety Governance in South Korea: A Structured Narrative Review Using the FAO/WHO National Food Control System Framework

**DOI:** 10.3390/foods15122055

**Published:** 2026-06-06

**Authors:** Hao Shen, Jingqiu Ma, Lu Liu, Peiqi Lu, Congyu Lin, Qian Yang

**Affiliations:** 1 School of Humanities and Social Sciences, Yanbian University, Yanji 133002, China; shenhao0826@126.com; 2 School of Food Science and Technology, Jiangnan University, Wuxi 214122, China; majingqiu1224@163.com (J.M.); 17645800680@163.com (L.L.); 6230111208@stu.jiangnan.edu.cn (P.L.); 3 School of Life Science and Technology, Harbin Institute of Technology, Harbin 150006, China

**Keywords:** institutional integration, risk-based governance, food safety governance, South Korea, FAO/WHO framework, national food control system

## Abstract

South Korea is a highly import-dependent food economy and therefore offers a useful case for examining how an integrated national food control system can be built under trade openness, limited domestic agricultural capacity and changing consumer risk perceptions. This article presents a structured narrative review, rather than a causal impact evaluation, of South Korea’s transition from multi-agency food safety regulation toward an integrated, risk-based food control system. The review is organized through the FAO/WHO national food control system framework and maps Korean legal, institutional and operational evidence onto six analytical dimensions: legal foundations, institutional coordination, risk-based official controls, import supervision, traceability and recall, and risk communication. Examples of embedded risk-analysis principles include the Positive List System for pesticide residues with a default limit of 0.01 mg/kg for substances without a Korean MRL, inspection orders and risk-ranked import controls, barcode-linked recall blocking through the Hazardous Food Sales Prevention System, and public disclosure of unsafe directly purchased overseas products. Quantitative evidence is used descriptively: Korea’s agricultural and food imports reached USD 45.3 billion in 2024, hepatitis A notifications fell from 17,598 in 2019 to 3989 in 2020 after the salted-clam outbreak, and MFDS reported that 12 of 544 overseas direct-purchase products tested in the first half of 2020 contained restricted substances. These indicators suggest improvements in coordination and crisis response capacity, but they do not prove that institutional integration alone reduced foodborne disease incidence. The review finds that South Korea’s model is strongest in institutional consolidation, import-oriented technical standards and digital recall communication, while key challenges remain in small-business compliance burden, scientific independence, data transparency, cross-border e-commerce and novel foods such as cell-cultured food ingredients.

## 1. Introduction

South Korea’s food system is characterized by limited arable land, high urbanization, diversified consumption and substantial exposure to imported food and agricultural commodities. Recent trade data underline the scale of this exposure: the Republic of Korea imported USD 45.3 billion of food and agricultural products in 2024, with consumer-oriented products representing the largest category. In food security debates, South Korea is often described as having a particularly low grain self-sufficiency rate; public sources report that the grain self-sufficiency rate dropped below 20%, while rice remains much more self-sufficient than wheat, corn or soybeans [1,2]. Import dependence makes food safety governance not only a domestic public health question but also a trade, standards and supply-chain-resilience question.

For this reason, “food safety governance” is defined here as a multidimensional system comprising: (1) legal coherence and enforceable standards; (2) institutional coordination among central ministries, local governments and laboratories; (3) risk assessment, risk management and risk communication; (4) official controls for domestic production, imports, distribution and food service; (5) traceability, recall and incident response; (6) compatibility with international trade rules; and (7) the capacity of businesses and consumers to comply with and use safety information. This definition is narrower than general food policy and broader than pathogen control alone.

The international rules surrounding food safety have shaped Korean reform. Under the WTO Agreement on the Application of Sanitary and Phytosanitary Measures, food safety measures should be based on scientific principles and, where possible, international standards; under the WTO Agreement on Technical Barriers to Trade, technical regulations and conformity-assessment procedures should avoid unnecessary obstacles to trade [3,4]. Codex Alimentarius standards, guidelines and recommendations therefore have practical importance in maximum-residue limits, contaminant standards, labeling and risk-analysis procedures [5]. Korea’s reforms can be understood as a process of external rule alignment and domestic adaptation: international rules created incentives for science-based standards and transparency, while domestic pressures—import dependence, food scandals and consumer distrust—created incentives for institutional consolidation and fast incident response.

Before the creation of the Ministry of Food and Drug Safety (MFDS) as a ministry-level authority in 2013, Korean food safety responsibilities were dispersed across several agencies and policy domains. Earlier studies described this as a fragmented system in which agricultural production, livestock products, processed foods, imports and food service were not always governed through the same information loop [6,7]. The establishment of MFDS did not eliminate the roles of the Ministry of Agriculture, Food and Rural Affairs (MAFRA), the Ministry of Oceans and Fisheries (MOF), local governments or quarantine bodies, but it did create a more visible central authority for standards, import control, inspection, recall and public risk communication.

However, the literature has not yet provided a fully evidence-based assessment of the Korean model. Many studies explain Korean laws or specific standards, while others focus on Codex harmonization, functional foods, residue monitoring or comparative lessons for China. Few studies combine institutional architecture, risk-based operations and outcome indicators in a structured way; fewer still distinguish descriptive association from causal evaluation. To make this gap explicit, Table 1 synthesizes representative prior studies and official reviews relevant to South Korea and comparable food control systems.

This study addresses three research questions: (1) How has South Korea organized institutional integration in national food control after the 2013 reform, and what remains outside a fully centralized model? (2) How do Korean risk-based instruments—such as PLS, import risk targeting, digital traceability and recall communication—map onto the FAO/WHO national food control system framework? (3) What descriptive quantitative evidence is available on system inputs, enforcement processes and public health outcomes, and what evidentiary limits prevent strong causal claims?

## 2. Evaluation Framework and Methodology

### 2.1. Review Type and Analytical Strategy

This article is revised as a structured narrative review with policy-analysis and evidence-mapping components. It is not presented as a full FAO/WHO diagnostic mission, a quantitative impact evaluation, or a qualitative comparative analysis (QCA). The FAO/WHO framework is used to structure evidence and identify gaps, while the empirical material is analyzed through legal-institutional interpretation, descriptive statistics and comparative policy analysis.

The distinction is important. The review can evaluate whether South Korea has the institutional elements and operational tools associated with a modern food control system; it cannot, without microdata and counterfactual modeling, determine whether the 2013 institutional reform independently caused reductions in foodborne illness. Therefore, all outcome-related statements are formulated as descriptive associations or plausible contributions rather than definitive causal effects.

### 2.2. Sources, Search Strategy and Inclusion Criteria

Sources were selected from four groups. First, official framework documents were consulted, especially the FAO/WHO Guidelines for National Food Control Systems and the FAO/WHO Food Control System Assessment Tool [14,15]. Second, Korean legal and administrative sources were reviewed, including English translations of statutes and MFDS, MAFRA, KDCA and FoodSafetyKorea materials where available. Third, scientific literature was searched in Web of Science, Scopus, PubMed, Google Scholar, KCI and RISS using English and Korean terms. Fourth, international comparator sources were drawn from the WTO, Codex, FDA, EFSA, OECD, USDA GAIN and official legal portals.

Search terms included combinations of English keywords such as “South Korea food safety governance”, “MFDS food control system”, “Korea Positive List System”, “Korea imported food safety”, “food traceability Korea”, “FoodSafetyKorea recall”, “HACCP Korea small business”, “Korea cultured food”, and “hepatitis A salted clams Korea”. In addition, Korean-language searches were conducted using original Korean terms corresponding to “food safety management system”, “Ministry of Food and Drug Safety”, “imported food safety management”, “Positive List System for pesticides”, “food traceability management”, and “overseas direct-purchase foods” in order to retrieve domestic legal, policy, administrative, and public health materials. The search emphasized the period from 2000 to 2025, while earlier legal and institutional sources were retained when they were foundational to the development of Korea’s food safety governance system.

Inclusion criteria were: (1) direct relevance to Korean food safety law, institutions, official control, imports, residue standards, traceability, recall, epidemiology or risk communication; (2) official or peer-reviewed source status, or clearly identified industry evidence used only as illustrative material; (3) traceable bibliographic or web information; and (4) sufficient specificity to support the claim being made. Exclusion criteria were: (1) papers on packaging films, polymers, environmental exposure, general nutrition or unrelated Korean social policy unless directly connected to food control; (2) sources that mentioned “Korea” but did not address food safety governance; and (3) sources whose data could not be traced to an official or published record.

Korean-language legal and government documents were included when relevant. Statutory titles and institutional names were translated into English for readability; the legal meaning was checked against official English translations when available. Where only Korean documents existed, the study reports high-level findings and avoids over-specific claims that could not be verified by an international reader.

### 2.3. Indicator Mapping to the FAO/WHO Framework

To better align the indicators with relevant FAO/WHO assessment elements, data sources and limitations, Table 2 maps and analyzes the indicators, data sources and reference years used in this review.

### 2.4. Data Extraction and Quantitative Treatment

Data were extracted into a structured scheme containing: source type, issuing body, year, jurisdiction, regulatory domain, indicator, numerator/denominator when available, and stated limitation. Quantitative indicators were used for descriptive support only. No interrupted time-series model, difference-in-differences design or confounder-controlled epidemiological model was conducted because the publicly available series are incomplete and definitions of outbreaks, inspections and enforcement actions differ across agencies and years.

For outcome interpretation, the review separates three evidence levels. Level 1 is legal or institutional evidence showing that a mechanism exists. Level 2 is operational evidence showing that a mechanism has been used, such as inspection orders, recall notices or overseas direct-purchase restrictions. Level 3 is outcome evidence, such as changes in cases or outbreaks. Only Level 3 evidence can support claims about public health performance, and even this does not establish causality without additional modeling.

### 2.5. Methodological Limitations

Four limitations should be emphasized. First, government-reported data may understate non-compliance if inspection intensity changes or if businesses alter behavior in anticipation of inspection. Second, epidemiological records suffer from under-reporting, changes in case definitions and different outbreak-attribution methods. Third, cross-country comparison is limited because the United States, European Union, China and South Korea divide risk assessment, risk management and local enforcement differently. Fourth, not all Korean datasets are available in English or in machine-readable formats; therefore, this review prioritizes traceable official sources and avoids unsupported numerical claims.

These limitations are not treated as minor caveats. They define the evidentiary boundary of the article: South Korea’s institutional integration may have contributed to better coordination and faster crisis response, but its independent effect on population-level foodborne disease incidence requires future quantitative evaluation using harmonized inspection, recall and epidemiological microdata.

## 3. Institutional Architecture of South Korea’s Food Control System

### 3.1. From Fragmented Administration to Coordinated Central Authority

Prior to the 2013 ministerial restructuring, Korean food safety regulation was distributed among ministries responsible for health, agriculture, fisheries, livestock products, imports, local food service and consumer protection. Fragmentation became visible during import-related scares and recall episodes, including the 2008 melamine-contaminated dairy-products crisis and recurrent alerts concerning imported foods. These episodes exposed three structural weaknesses: dispersed responsibility for upstream and downstream controls, delayed information sharing across agencies, and inconsistent public communication.

**Figure 1:**
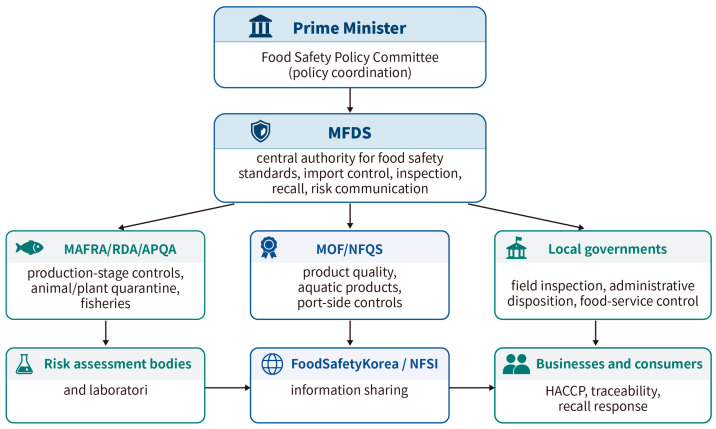
Institutional architecture of South Korea’s integrated food safety governance. The figure emphasizes that MFDS is the central food-safety authority but that production-stage controls, fisheries, local enforcement, laboratories, information systems, businesses and consumers remain connected through a matrix rather than a fully centralized hierarchy.

The 2013 establishment of MFDS as a ministry-level institution created a more coherent center for food and drug safety policy. MFDS now exercises core functions in food standards, imported food safety, food sanitation, recall, labeling, health functional foods and risk communication. However, the Korean model should not be described as complete centralization. MAFRA retains important responsibilities over agricultural production, animal disease control and plant/animal quarantine; MOF and fisheries bodies retain aquatic-product functions; local governments conduct many front-line inspections; and specialized laboratories and information agencies provide technical support. The result is a matrix-style governance structure rather than a single vertical command chain (Figure 1).

Matrix-style governance means that authority is organized simultaneously by product stage, hazard type and administrative level. For example, pesticide residue limits may be set and managed through MFDS food standards, while farm-level pesticide use involves agricultural authorities; imported foods are controlled at the border by MFDS systems, but origin-country compliance depends on foreign producers and sometimes exporting-country certification; recalls are nationally disclosed through FoodSafetyKorea but executed through businesses, retailers and local authorities.

### 3.2. Legal Foundations and Their Functions

To better align the indicators with relevant FAO/WHO assessment elements, data sources and limitations, Table 3 maps and analyzes the indicators, data sources and reference years used in this review.

### 3.3. Institutional Resources and Enforcement Context

Institutional resources are important but difficult to compare internationally because agencies differ in mandate. The original manuscript used a limited resource table with a draft caption; the revised version treats resource figures as input indicators and avoids inferring effectiveness directly from budget or staff size. Table 4 combines input indicators and contextual figures available from official or traceable sources. Where exact annual enforcement series are not publicly harmonized, the table reports only indicators that can be tied to an identifiable source.

Enforcement effectiveness depends on legal powers, laboratory capacity, risk targeting, local implementation and business response. South Korea’s stricter standards, especially the PLS, can create market-access frictions for exporters when no Korean MRL or import tolerance exists. However, such frictions are not automatically “trade disputes.” Under the SPS framework, they become more problematic when measures are not transparent, not science-based or discriminatory. Korea has therefore expanded import-tolerance procedures and information tools to reduce avoidable disruption while maintaining a protective default threshold.

## 4. Operational Mechanisms: Risk-Based Monitoring, PLS, Traceability and Crisis Response

### 4.1. Risk-Oriented Targeted Monitoring and Import Controls

**Figure 2:**
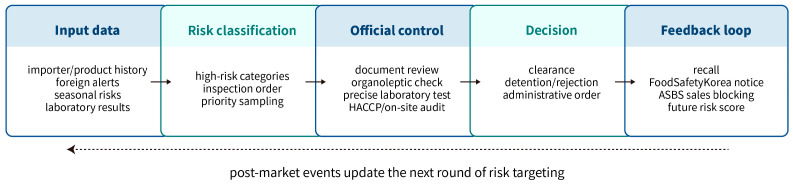
Risk-based operational loop for South Korea’s food control system. The exact algorithmic weights used by Korean authorities are not public; the figure therefore shows the policy logic rather than a verified scoring model.

South Korea’s operational system has moved from conventional end-product inspection toward risk targeting (Figure 2). In import control, MFDS combines document review, organoleptic examination, precise laboratory testing, random sampling and inspection orders. Input data may include product type, exporting country, manufacturer history, previous non-compliance, international risk alerts and seasonal hazard patterns. The precise risk-scoring algorithm and inspection frequency by product category are not fully disclosed in public English sources; therefore, this review describes the mechanism without claiming exact frequencies not supported by accessible data.

The key analytical point is that risk-based inspection seeks to allocate laboratory resources toward products and sources with higher expected risk rather than treating all consignments equally. This is consistent with FAO/WHO guidance, which emphasizes risk-based official controls, laboratory support, transparent prioritization and feedback from surveillance results. The policy value lies not merely in stricter testing but in closing the feedback loop: non-compliance changes future risk classification, which can trigger stronger inspection orders or import restrictions.

### 4.2. Positive List System and Maximum-Residue Governance

The Positive List System (PLS) is one of the most important technical elements of Korea’s risk-based food control model. For pesticide residues, Korea fully implemented the PLS for all agricultural products from 1 January 2019 after earlier application to nuts, seeds and tropical fruits [24]. Under this system, when no Korean MRL or import tolerance is established for a pesticide/commodity combination, a default limit of 0.01 mg/kg (0.01 ppm) applies [25,26]. For veterinary drugs, Korea has also moved toward a PLS approach for animal-origin products; USDA reporting indicates that, in the absence of a Korean MRL or import tolerance, a 0.01 ppm residue tolerance applies under the veterinary drug PLS schedule [27].

The scope should therefore be stated carefully. The agricultural PLS concerns pesticide residues in agricultural products, while the veterinary-drug PLS concerns animal-origin products and follows a separate implementation pathway. They share the technical logic of a positive list and restrictive default threshold, but they are not identical programs.

Internationally, Korea’s pesticide PLS resembles Japan’s positive list system, introduced in 2006, and the European Union’s practice of applying a default low MRL when no specific MRL is set (Table 5) [28]. The Korean system is distinctive in its import-dependent context and in the policy salience of import tolerances. Exporters must verify Korean MRLs rather than assuming that Codex or another country’s tolerance will apply automatically. This has practical consequences for products such as tropical fruits. During the early implementation stage, banana exporters to Korea raised concerns that many recognized chemicals for bananas would become subject to the 0.01 ppm threshold unless specific MRLs or import tolerances were established [29]. This example illustrates how the PLS changes supply-chain behavior: exporters must collect residue data, seek import tolerances, modify pesticide-use practices or segregate compliant lots before shipment.

The PLS should not be described only as a trade barrier. It is also a risk-management tool that reduces ambiguity when no compound-specific standard exists. At the same time, the system requires transparent MRL databases, reasonable import-tolerance procedures and communication with trading partners to prevent disproportionate disruption for lower-risk products.

### 4.3. Hepatitis A Outbreak: Useful Case but Not Causal Proof

**Figure 3:**
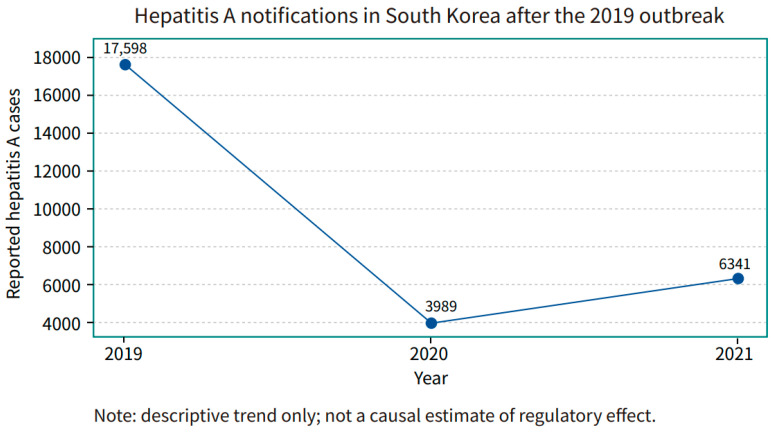
Reported hepatitis A cases in South Korea after the 2019 salted-clam outbreak. Data source: KDCA surveillance data [30]. The figure reports descriptive surveillance counts only; the reduction should not be interpreted as a causal estimate of regulatory intervention.

The 2019 hepatitis A outbreak linked to salted clams provides a useful case for examining crisis response because it involved epidemiological investigation, product tracing, recall, public communication, and consumer-behavior change. Korean public health reporting recorded a sharp increase in hepatitis A notifications in 2019, with 17,598 cases, followed by 3989 in 2020 [30]. Figure 3 presents this descriptive change in reported cases. However, this decline should not be interpreted as direct causal proof that regulatory intervention alone reduced hepatitis A incidence. Rather, it indicates that the outbreak response was temporally associated with investigation, product recalls, risk communication, and subsequent monitoring. Other factors, including vaccination, changes in reporting practices, sanitation conditions, consumer behavior, and COVID-19-related social changes, may also have contributed to the observed decline.

Thus, the outbreak demonstrates the value of a coordinated traceability and communication system, not proof that institutional integration alone reduced hepatitis A incidence. Future research should use case-level data, outbreak timelines, vaccination coverage and food-consumption data to estimate causal effects.

### 4.4. Digital Traceability, Recall and Privacy Safeguards

Korea has invested in traceability and recall communication through FoodSafetyKorea, the National Food Safety Information Service and the Hazardous Food Sales Prevention System [31]. The latter links recall information to retail point-of-sale systems so that recalled products can be blocked through barcode scanning. Such tools shorten the information chain among regulators, retailers and consumers, especially when the product is widely distributed.

The original manuscript used the phrase “second-level traceability” without sufficient technical evidence. This revision removes the unsupported claim and replaces it with verifiable functions: product-level recall disclosure, barcode-linked sales blocking, traceability requirements for specified categories, and public release of unsafe product information. Exact recall-time improvements and system coverage by product category should be treated as future empirical questions unless official performance series become available.

Digital traceability also raises data-governance concerns. Personal data in Korea is governed by the Personal Information Protection Act, and public information systems are subject to public-sector cybersecurity and access-control rules [32]. However, the detailed cybersecurity architecture of food traceability platforms is not fully public. The appropriate conclusion is that legal safeguards exist, but independent audit evidence on privacy, interoperability and cyber resilience remains limited.

## 5. International Perspective: United States, European Union and China

### 5.1. Korea and the United States FSMA Model

The comparison with the United States should be more precise than in the original manuscript. The U.S. Food Safety Modernization Act (FSMA) strengthened preventive controls, produce safety standards and FDA mandatory recall authority, as well as foreign supplier verification requirements [33,34]. It places substantial obligations on private firms, including hazard analysis, preventive controls, supply-chain programs and records [33,34]. Korea also emphasizes prevention and import controls, but its model is more visibly built around a central ministry, technical residue standards and border inspection tools.

The difference is therefore not simply that the United States is preventive and Korea is integrated. Both are preventive in different ways. FSMA relies heavily on firm-level preventive-control obligations and FDA oversight, while Korea combines centralized public authority, import inspection, PLS thresholds, HACCP accreditation and rapid public recall communication.

### 5.2. Korea and the EU Risk-Assessment Model

The European Union separates scientific risk assessment from risk management more explicitly through the European Food Safety Authority (EFSA), the European Commission and member-state competent authorities [33,34]. The main differences in governance structures and enforcement mechanisms across selected countries and regions are shown in Table 6 [35,36]. Korea’s MFDS model integrates many risk-management and communication functions within one ministry. This can improve speed and administrative coherence, especially in crises. It also creates a governance risk: if scientific assessment, policy management and public communication are too closely bundled, perceived independence may be weakened.

Accordingly, the Korean model would be strengthened by more transparent publication of assessment protocols, conflict-of-interest rules, advisory committee membership, data-quality assessments and uncertainty statements. Scientific independence does not require copying EFSA’s institutional design, but it does require visible safeguards separating evidence assessment from political or administrative pressure.

## 6. Practical Challenges and Emerging Issues

### 6.1. SME Compliance Burden and Proportional Regulation

SME compliance burden is a genuine challenge. HACCP, traceability, residue testing, documentation and digital reporting are not costless, and smaller processors often lack specialized staff. MFDS recognizes this problem by providing customized technical support, training and promotion for small businesses through the Korea Institute for Food Safety Management Accreditation. However, Korea-specific public survey data on the direct cost of compliance remain limited. International studies of small and medium-sized food businesses show that compliance costs, documentation time and technical expertise are common barriers, suggesting that Korea should combine mandatory standards with proportional tools for lower-risk micro-enterprises [37,38].

Policy options include simplified hazard-control templates, subsidized laboratory testing for small firms, shared digital traceability services, phased implementation for lower-risk categories and targeted training. The aim should be “same safety objective, differentiated compliance pathway,” not reduced safety for small firms.

### 6.2. Novel Foods and Cell-Cultured Food Ingredients

Novel foods require more precise regulatory discussion. In 2024, MFDS listed Standards for Recognition of Temporary Standards and Specifications for Foods, which created a pathway relevant to cell- and microbial-culture food ingredients [39]. Recent research comparing cultured-food safety evaluation systems also notes that Korea evaluates cell-cultured food ingredients through a novel-food-style recognition system [40]. The regulatory issues are not limited to whether cultured meat is “safe” in general. They include cell-line provenance, culture media and scaffold residues, allergenicity, genetic stability, microbial contamination, antibiotic or growth-factor residues, nutritional equivalence, labeling, post-market monitoring and consumer communication. A regulatory sandbox may be useful, but it should be paired with transparent dossier requirements, public summaries of safety decisions and post-market surveillance.

### 6.3. Cross-Border e-Commerce and Overseas Direct Purchases

Cross-border e-commerce challenges are no longer hypothetical. MFDS has reported testing overseas direct-purchase products and requesting customs withholding when harmful substances are found [41]. In the first half of 2020, MFDS bought 544 overseas online products advertised for weight loss or sexual-function improvement and reported that 12 products contained restricted substances; it restricted 128 hazardous or potentially hazardous products [42]. This illustrates the same regulatory problem: products can reach consumers through individual parcel channels before ordinary importer controls apply.

The regulatory solution requires platform cooperation, customs data linkage, rapid public lists of unsafe products, consumer education and international information exchange. Because many products are marketed as “supplements” or functional foods, border control should be linked with advertising and health-claim enforcement.

### 6.4. Risk Communication, Social Media and Nutritional Implications

Risk communication should be treated as a core component of food control rather than a secondary public-relations issue. Two examples show why. First, public anxiety over Japanese seafood and Fukushima treated-water discharge generated salt hoarding and reduced seafood demand in South Korea [38], as well as public protests against Japan’s treated-water release plan [43], even as official monitoring and scientific explanations continued. Second, the 2024 social media trend of frying starch toothpicks forced MFDS to warn that toothpicks are sanitary products, not foods, and that their safety as food had not been verified [44]. Both cases show that social media can convert uncertainty or novelty into consumption behavior faster than ordinary regulatory notices can respond.

The integrated system also affects nutrition only indirectly. Safer imports, residue controls and labeling can support dietary diversity and reduce exposure to pathogens, toxic residues or misleading health claims. However, the reviewed evidence does not show that institutional integration directly reduced chronic diseases or nutritional deficiencies. Claims about nutrition should therefore be framed as plausible pathways—safe supply, truthful labels, and protection of vulnerable consumers—rather than measured health outcomes.

## 7. Conclusions

This revised review concludes that South Korea has built a relatively advanced, import-sensitive and risk-oriented food safety governance model centered on MFDS but supported by a wider matrix of agricultural, fisheries, local government, laboratory, information and private-sector actors. Its strongest features are institutional consolidation after 2013, science-based residue governance through the PLS, risk-targeted import controls, digital recall communication and public disclosure of hazardous overseas direct-purchase products.

The conclusions are deliberately more cautious than in the original manuscript. Descriptive evidence suggests that institutional integration may have improved regulatory coordination and crisis response capacity, but available public data do not justify a claim that the reform alone significantly reduced foodborne disease frequency. The hepatitis A case illustrates the capacity for traceability and communication, but it also demonstrates the need to control for vaccination, sanitation, consumer behavior and reporting changes.

The article’s contribution lies in making the literature gap explicit, mapping Korean governance instruments to the FAO/WHO framework, removing irrelevant citations, and identifying the data needed for a future system assessment: harmonized annual series on inspections, non-compliance, administrative actions, border detentions, recalls, recall completion times, foodborne outbreaks and hazard-specific monitoring results. Future research should develop a transparent FAO/WHO compliance matrix with scoring criteria and should test causal claims through time-series or comparative designs. For policymakers, the Korean experience suggests that integrated authority is valuable only when paired with transparent evidence, independent scientific review, proportional compliance support and credible public communication.

## Figures and Tables

**Table 1:** Synthesis of prior literature and remaining gaps in Korean and comparable food safety governance research.

Study	Main Focus	Methodology	Scope	Gap Left Unaddressed
Sohn and Oh, 2013/2014 [6]	Institutional reform, harmonization and automatic product recall	Policy review/perspective	Korea, mostly 2008–2013	Highlights MFDS consolidation and ICT recall systems but does not provide a full FAO/WHO indicator matrix or causal outcome analysis
Lee et al., 2021 [7]	Codex, Korean standards and food safety management	Review of Codex harmonization	Korea and Codex, contemporary system	Strong on standards alignment; limited on enforcement data, local implementation and epidemiological outcomes
Lee, 2014 [8]	Agro-food safety control structure	Descriptive institutional review	Korea, pre/post-2013 transition	Useful institutional background; limited quantitative validation and no explicit risk-based performance assessment
Jianjun, 2016 [9]	Food safety standards system of South Korea	Legal/standards review	South Korea, standards system	Focuses on standards architecture; does not assess operational effectiveness or outcomes
Ningxin et al., 2014 [10]	Korean food safety laws and implications for China	Comparative legal review	South Korea and China	Provides comparative lessons but remains policy-descriptive and not data-driven
Son, 2023 [11]	Public food procurement and food policy transition	Policy-transition analysis	South Korea, local and national procurement	Relevant to food governance but not primarily a food safety control-system evaluation
Kim et al., 2025 [12]	Veterinary drug residues in livestock products	Monitoring and risk assessment	Republic of Korea, long-term monitoring	Provides residue-risk data but focuses on one hazard domain rather than institutional governance
Holdaway and Husain/FORHEAD, 2013 [13]	Food safety governance in China	Cross-sector mapping review	China, multi-sector risks	Useful comparator for integrated governance; not Korea-specific and illustrates the need for data integration

**Table 2:** Mapping of FAO/WHO assessment elements to indicators, sources and limitations used in this review.

FAO/WHO Element	Indicator Used in This Review	Data Source	Years	Data Type	Limitations
Policy and legal framework	Existence and scope of framework laws, food sanitation law, imported food law, health functional food law, labeling law [16,17,18,19,20,21]	Korean legal database; MFDS English regulations; National Assembly records	2000–2025	Normative/legal	Legal existence does not show enforcement quality; translations may lag behind Korean amendments
Institutional capacity and coordination	MFDS mandate, regional offices, inter-ministerial coordination, division of roles with MAFRA/MOF/local governments	MFDS website; laws; annual reports; official organizational charts	2013–2025	Institutional/descriptive	Budget and staffing categories are not always comparable across ministries or countries
Risk-based official control	Inspection orders, risk categorization, PLS, HACCP support, targeted import checks [22,23]	MFDS Imported Food Safety pages; USDA GAIN; KREI; WTO SPS materials	2017–2025	Process/administrative	Public sources rarely disclose the exact algorithm or inspection-frequency weights
Laboratory and surveillance support	Residue monitoring, veterinary drug residue assessment, laboratory testing and foreign testing laboratory designation	MFDS; peer-reviewed residue monitoring studies; official notices	2013–2025	Technical/quantitative	Hazard-specific monitoring does not represent all food safety risks
Traceability, recall and communication	FoodSafetyKorea recall disclosure, hazardous-food sales blocking, overseas direct-purchase product warnings	MFDS; FoodSafetyKorea; NFSI; official press releases	2009–2025	Process/outcome-adjacent	Technical recall-time data and cybersecurity architecture are not fully public
Outcome and public health indicators	Hepatitis A notifications, waterborne/foodborne outbreak reports, recall/non-compliance examples	KDCA; official epidemiological reports; peer-reviewed outbreak studies	2013–2024	Epidemiological/descriptive	Changes may reflect sanitation, vaccination, reporting behavior, COVID-19 measures and consumer behavior, not regulation alone

**Table 3:** Major legal instruments supporting South Korea’s food control system.

Legal Act	Scope	Responsible Authority	Importance for Food Control	Relationship with Codex/WTO
Framework Act on Food Safety	National food safety policy principles and coordination; basis for policy committees and cross-ministerial planning	Prime Minister/central and local authorities	Provides whole-of-government coordination logic	Supports science-based and risk-management approaches consistent with Codex/WTO principles
Food Sanitation Act	General food hygiene, business licensing, safety standards, inspection, administrative disposition and recall	MFDS and local governments	Core statute for everyday food sanitation and enforcement	Implements domestic sanitary controls and food standards
Special Act on Imported Food Safety Control	Registration, inspection, traceability and control of imported foods, including risk-based inspection and inspection orders	MFDS	Central statute for import-dependent food risk management	Operationalizes SPS-compatible border controls and import tolerance management
Health Functional Foods Act	Safety, efficacy-related claims, manufacturing and labeling of health functional foods	MFDS	Important for high-growth supplement market and cross-border claims	Requires evidence-based functionality and labeling controls
Livestock Products Sanitary Control Act	Hygiene and safety of livestock products; slaughtering, processing and distribution controls	MFDS, MAFRA/APQA and local authorities	Addresses animal-origin food hazards and veterinary drug residues	Connects food hygiene with animal health and residue standards
Act on Labeling and Advertising of Foods	Labeling, advertising, nutrition information and misleading claims	MFDS and local governments	Supports consumer information and risk communication	Relevant to TBT transparency and truthful labeling

**Table 4:** Institutional resource and operational context indicators for South Korea’s food safety governance.

Indicator	Value/Description	Year	Source Type	Interpretive Limitation
MFDS personnel	1946 employees	2020	Original MFDS/World Bank-reported administrative indicator cited in the previous manuscript; retained only as input context	Not comparable to FDA/EFSA because mandates differ
MFDS operating budget	Approximately KRW 540 billion	2020	Administrative input indicator used in the previous manuscript	Budget categories do not isolate food safety from drug/medical-product functions
MFDS regional food and drug safety administrations	6 regional offices	2024–2025	MFDS organizational information	Indicates subnational coverage but not inspection intensity
Agricultural and food imports	USD 45.3 billion; consumer-oriented products USD 20.6 billion	2024	USDA GAIN Exporter Guide Annual	Trade value indicates exposure but not risk level
Hazardous Food Sales Prevention System	Implemented through barcode-linked blocking at more than 40,000 retail stores as reported by Sohn and Oh	2009 onward	Peer-reviewed policy review	Coverage and recall-time performance require updated official series
Overseas direct-purchase inspection	544 products purchased/tested; 12 contained restricted substances; 128 hazardous or potentially hazardous products restricted	First half of 2020	MFDS press release	Targeted sampling cannot estimate prevalence among all overseas purchases

**Table 5:** Comparison of Korea’s PLS with Japanese and EU residue-limit approaches.

Jurisdiction	System	Default Approach	Implementation Context	Relevance for Korea Comparison
Korea	Pesticide PLS; veterinary-drug PLS developing separately	0.01 mg/kg/ppm when no Korean MRL or import tolerance exists	Nuts/seeds and tropical fruits from 2017; all agricultural products from 2019; veterinary-drug PLS schedule from 2024	Strong import-tolerance relevance; exporters must check Korean MRL database
Japan	Positive list system for agricultural chemical residues	Generally 0.01 ppm for substances without a specific standard	Implemented in 2006	Longer implementation history; similar exporter compliance burden
European Union	MRL system under Regulation (EC) No 396/2005 [28]	Default 0.01 mg/kg unless another MRL applies	Current EU-wide system	EFSA risk assessment and EU risk management are institutionally separated

**Table 6:** Comparative governance structures and enforcement mechanisms in selected food control systems.

Jurisdiction	Governance Structure	Risk Profile	Regulatory Focus/Mechanism	Analytical Implication
South Korea	MFDS-centered integrated ministry with roles for MAFRA, MOF and local governments	Import dependence, residues, processed foods, e-commerce, public trust	Central standards, import inspection, PLS, HACCP, FoodSafetyKorea, recall blocking	Efficiency and fast communication; risk of limited visibility of scientific independence
United States	FDA-centered preventive controls with USDA roles for meat/poultry/eggs	Large domestic market plus imports; firm-level preventive control	FSMA preventive controls, FSVP, mandatory recall, facility records [33,34]	Strong private-sector obligations; fragmented federal mandate remains
European Union	EFSA risk assessment, European Commission/member-state risk management [35,36]	Internal market, cross-border trade, residue and contaminant harmonization	RASFF, EU MRLs, official controls regulation, separation of assessment/management	Scientific independence more visible; multi-level coordination can be slower
China	State Administration for Market Regulation and sectoral agencies; local government responsibility	Scale, fragmented production, e-commerce, imported foods, local enforcement disparities	Food Safety Law, national standards, risk monitoring, platform governance	Useful comparator for integrated-market supervision, but local implementation varies

## Data Availability

No new data were created or analyzed in this study. Data sharing is not applicable to this article.
